# Suspected Primary Spontaneous Asymptomatic Pneumothorax in a Cat

**DOI:** 10.1155/2022/2827118

**Published:** 2022-02-14

**Authors:** Michael Sliman, Adam J. Rudinsky, Sarah Lumbrezer, Jenessa A. Winston, Valerie J. Parker, Sarah Lorbach, James Howard

**Affiliations:** ^1^Department of Veterinary Clinical Sciences, The Ohio State University, Columbus, OH, USA; ^2^Comparative Hepatobiliary and Intestinal Research Program, The Ohio State University, Columbus, OH, USA

## Abstract

Spontaneous pneumothorax (SPT) is a documented emergency of the respiratory tract condition classified as either primary or secondary based on the presence of underlying pulmonary conditions. All reported SPT in the feline literature are evaluated for respiratory clinical signs. Primary SPT without underlying pathology or without clinical signs is not reported in cats. This case report describes a 10-year-old domestic longhair cat that was referred for evaluation of chronic lethargy with severe azotemia and placement of a subcutaneous ureteral bypass (SUB) system. Prior to presentation, the cat was diagnosed with renal insufficiency and treated medically with no resolution. Clinical examination under sedation revealed right-sided renomegaly. Thoracic radiographs revealed gas in the caudodorsal pleural space and concurrent pulmonary atelectasis. No respiratory clinical signs were present. Thoracic CT showed two pulmonary bullae, one located in the right caudal lung lobe and one in the cranial segment of the left cranial lung lobe. Abdominal ultrasound showed a right-sided ureteral obstruction. Medical management was elected for the spontaneous pneumothorax. A SUB was placed to address the ureteral obstruction; no complications were noted during recovery. The cat was free of clinical signs of respiratory disease after a follow-up time of nine months. This is the first reported case of a cat diagnosed with a nonclinical suspected primary spontaneous pneumothorax with no concurrent predisposing pulmonary pathology.

## 1. Introduction

Pneumothorax is a pathologic condition where atmospheric air enters the pleural space causing the lungs to collapse secondary to loss of negative intrathoracic pressure within the pleural space [1]. This loss of negative intrathoracic pressure decouples the visceral and parietal pleura resulting in variable degrees of atelectasis. Depending on the cause of the pneumothorax and severity of the respiratory compromise, this condition can result in respiratory distress secondary to pulmonary insufflation inhibition. Compensatory mechanisms, such as hyperventilation, exaggerated chest wall excursions, and recruitment of abdominal musculature are used to normalize breathing patterns, tidal volume, and minute ventilation [2].

Pneumothorax can be categorized based on etiology: spontaneous, traumatic, and iatrogenic. Traumatic pneumothorax is the most common cause of pneumothoraces in small animal patients. Fifty percent of all traumatic chest injuries in dogs are classified as traumatic SPT [2, 3]. These can be caused by blunt force trauma, bite wounds, penetrating foreign bodies, projectiles (e.g., gun shots), lung lobe laceration, and iatrogenic causes (e.g., needle thoracocentesis), among others [2]. Spontaneous pneumothorax occurs when air enters the pleural cavity through defects in the pulmonary parenchyma, unrelated to a traumatic or iatrogenic etiology [4]. Traditionally, SPTs are categorized as either primary or secondary based on the presence of specific underlying pulmonary pathology. Primary SPTs result from pulmonary blebs or bullae rupturing in the absence of concurrent lung disease [4]. Secondary SPTs occur when there is an underlying pulmonary pathology associated with air accumulation in the pleural space [2]. Dogs are at a higher risk for developing primary SPT versus secondary, as it accounts for approximately 68% of all SPT cases in canine patients [3]. SPTs are rarely reported in cats with all reported causes due to secondary causes such as inflammatory airway disease [5–7], heartworm infection [8], *Aelurostrongylus abstrusus* infection [9], neoplasia [7], and bronchopulmonary dysplasia [10]. Most cats with SPT present with signs of acute respiratory distress including dyspnea, coughing, and abnormal lung sounds [11, 12]. Other clinical signs of cats diagnosed with SPT include ocular discharge, heart murmur, gallop rhythms, lingual ulceration, muffled heart sounds, tachycardia, bradycardia, cyanosis, and pale pink mucus membranes [11]. This report represents a distinct difference in case presentation as it describes the occurrence of a suspected primary SPT in a feline patient with no concurrent respiratory clinical signs or underlying pathology. This novel case report will describe the presentation, diagnosis, and treatment of a feline patient diagnosed with suspected primary SPT due to the rupture of pulmonary bullae/blebs in accordance with a modern translational approach used in people in which imaging modalities are used without the need for histopathology to diagnose primary SPTs.

## 2. Case Details

A 10-year-old 6.5 kg castrated indoor-only domestic longhair cat was presented to a tertiary referral specialty hospital for severe azotemia and no known comorbidities. Prior to presentation at the Ohio State Veterinary Medical Center, the cat was presented to its primary care provider for inappetence and vomiting. An initial biochemistry profile at that time showed severe azotemia with a creatinine of 9.7 (ref: 0.8-2.4 mg/dL) and blood urea nitrogen (BUN) of 113 (ref: 16–36 mg/dL). Abdominal radiographs subjectively showed asymmetrically sized kidneys with the left kidney described as extremely enlarged and the right kidney appearing small. Outpatient care was elected due to the cat's severe fractious behavior. The cat was medically managed with ondansetron, sucralfate, aluminum hydroxide, and subcutaneous fluids. Due to lack of improvement, the patient was represented two days after the initial onset of clinical signs to its primary veterinarian. On reevaluation, the patient's azotemia worsened, and the cat was referred to the Ohio State Veterinary Medical Center for further diagnostics and treatment.

On initial evaluation at the authors' institution, the cat was bright, alert, and responsive but extremely fractious. The patient was sedated for the remainder of the physical examination with no complications. Thoracic auscultation revealed no cardiovascular or respiratory abnormalities. The cat was estimated to be less than 5% dehydrated and an enlarged right kidney was documented. The remainder of the physical examination was unremarkable, and the decision was made to hospitalize the patient for initial diagnostics, supportive care, and overnight monitoring.

Initial diagnostics showed the cat's packed cell volume (PCV) and total protein (TP) were 30% (25-45) and 7.0 g/dL (6.1–8.0 g/dL), respectively. The venous blood gas revealed a blood pH of 7.12 (ref. 6.5-8.0 pH units), pCO_2_ of 34.5 (ref. 3.0-200 mmHg), blood urea nitrogen (BUN) > 100 (ref. 3-100 mg/dL), and a creatinine value exceeding the upper limits (12 mg/dL) of the analyzers' capabilities (ref. 0.2-12.0 mg/dL). The cat's complete blood count was largely unremarkable with a hematocrit (PCV) of 39% (ref. 2e5–45%), platelet count (PLT) of 173 × 10^9^ (ref. 128–444 × 10^9^/L), and total leukocyte count of 8.1 × 10^9^ (ref. 2.3–18.4 × 10^9^/L). The patient's biochemistry profile showed a BUN of 203 mg/dL (ref. 18–39 mg/dL), creatinine of 16.7 mg/dL (ref. 0.7-1.6 or 1.9 mg/dL), and phosphorous levels at 15.8 mg/dL (ref. 3.1-6.5 mg/dL). The urinalysis showed hyposthenuric urine with a specific gravity of 1.007. Repeat abdominal radiographs showed a right kidney at the upper limits of normal for size (5.4 cm long axis measurement) with a mineral opaque foci within the right renal pelvis and a small left kidney (3.5 cm long axis measurement) described as 1.7 times the length of L2 (normal range; 2.4 to 3.0 times the length of L2, older cats without signs of renal disease; 1.9 to 2.6 times the length of L2) [13, 14]. No other abnormal findings were noted. Treatment at this time included analgesics and fluid resuscitation with hourly monitoring.

**Figure 1:**
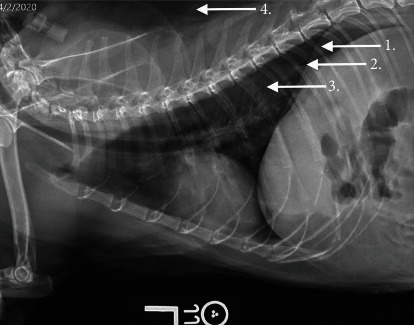
Thoracic radiographs showing gas accumulation in the caudodorsal pleura space (arrow 1), retraction of lung lobes (arrow 2), atelectasis and interstitial pattern (arrow 3), and subcutaneous emphysema (arrow 4) prominent on the left lateral view.

Physical examination and bloodwork the following day were largely static with the exception of a newly auscultated gallop rhythm. An echocardiogram was performed to evaluate cardiac function given the presence of the animal's gallop rhythm; findings were consistent with equivocal hypertrophic cardiomyopathy versus a normal variant. Abdominal ultrasound revealed an enlarged right kidney at 5.6 cm in length, bilateral pyelectasia (right renal pelvis measured at 5.7 mm, left renal pelvis measured at 10.3 mm), and proximal hydroureter (right ureter measured at 4 mm, left ureter measured at 4.1 mm) secondary to a ureteral obstruction, pyelonephritis, or ureteritis. Nodules were present within the left renal parenchyma. The heteroechoic nodule in the cranial pole measured 8.1 × 5.6 mm, and the similar nodule in the caudal pole measured 7.4 × 5.1 mm. Renal aspirates were performed, and cytology revealed rare mesenchymal cells, consistent either with normal stromal cells, reactive fibroplasia, scirrhous response, or possibly mesenchymal neoplasia. A hyperechoic structure, suspected to be biliary dystrophic mineralization or a cholelith, was also noted within a left liver lobe. Given the potential concern for renal neoplasia, three-view thoracic radiographs were performed. Thoracic radiographs revealed a moderate pneumothorax with intrapleural gas in the caudodorsal thoracic space causing retraction of the caudal lung lobes; this was most evident on the left lateral projection (Figure 1). There was a concurrent pulmonary atelectasis with patchy unstructured interstitial patterns in multiple lung lobes. Subcutaneous emphysema was also present consistent with previous subcutaneous fluid administration (Figure 1).

**Figure 2:**
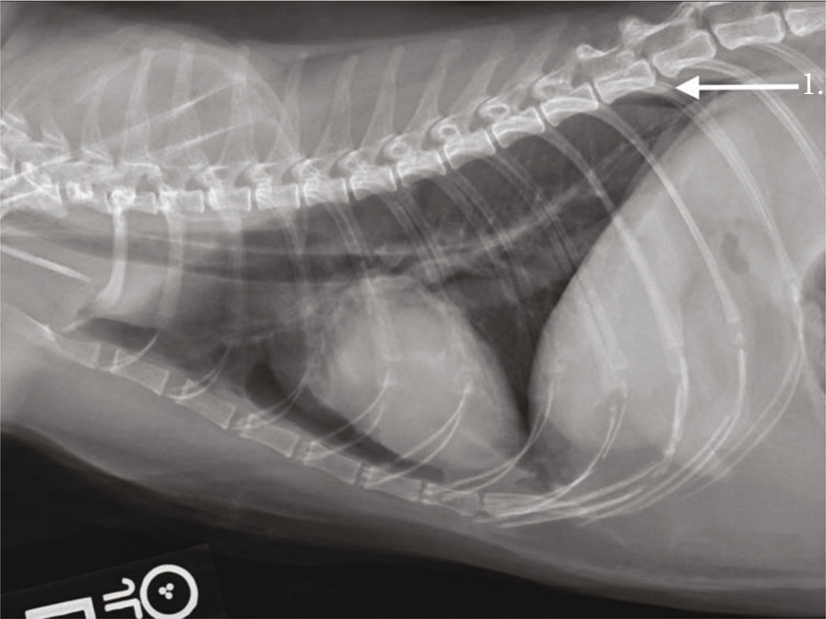
Left lateral view showing similar presence of gas in the pleural space (arrow 1).

**Figure 3:**
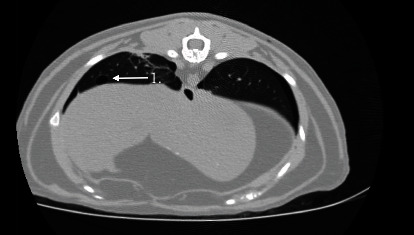
Right caudal lung lobe bulla in the lung window (arrow 1).

**Figure 4:**
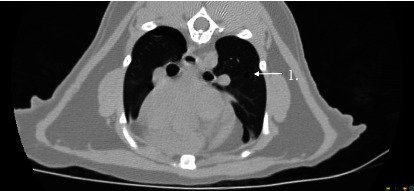
Cranial segment of the left cranial lung bulla in the lung window (arrow 1).

The following day the patient was placed under general anesthesia for repeat thoracic radiographs and thoracic CT prior to surgical placement of the bilateral subcutaneous ureteral bypass (SUB) system. The patient received midazolam (0.18 mg/kg IV) and methadone (0.27 mg/kg IV) for premedication and was induced with alfaxalone (0.77 mg/kg IV). Sevoflurane inhalant anesthesia was used to maintain general anesthesia. Thoracic radiographs showed a similar amount of gas within the pleural space and static concurrent pulmonary atelectasis prior to surgery. The subcutaneous emphysema was less severe (Figure 2). To further evaluate the underlying cause of the cat's pneumothorax, a thoracic CT was performed using a multidetector 64-slice CT (GE Revolution EVO, GE Healthcare, Waukesha, WI) with 1.25 mm slice thickness using pre- and postcontrast imaging sequences (Figures 3 and 4). A mild amount of gas persisted in the pleural space bilaterally, with the caudodorsal thorax most affected. Within the pulmonary parenchyma were two gas filled structures with a thin soft tissue rim, consistent with pulmonary bullae. One was located in the right caudal lung lobe and the other was in the cranial segment of the left cranial lung lobe. There were patchy, unstructured interstitial to alveolar patterns in all lungs, associated with atelectasis from the pneumothorax and general anesthesia. Incidental findings included mild subcutaneous emphysema, biliary mineralization, and a small gas focus in the cranioventral mediastinum, likely associated with the previous left jugular catheter placement. These findings indicated a spontaneous pneumothorax due to bilateral pulmonary bullae.

Due to the lack of clinically appreciable respiratory compromise, the patient did not undergo thoracic surgery. This decision was primarily based on the patient's severity of renal disease and concern for duration of anesthesia with tandem procedures. Reevaluation of the pneumothorax was recommended following its recovery from the SUB placement procedure and stabilization of the cat's azotemia.

Placement of the bilateral SUB was performed according to Norfolk Vet Products SUB surgical guide with no complications (*SUB™ 2.0 Surgical Guide*). An esophagostomy tube was placed following closure of the celiotomy with no complications. The general anesthetic event and surgical procedure were completed with no complications. The cat recovered under supervised care with no evidence of respiratory compromise or surgical complications.

The cat was hospitalized for five days for postoperative monitoring. Due to a declining PCV (16%) three days after surgery, the cat was transfused with packed red blood cells (6 ml/kg IV) with no complications. A follow-up urinary ultrasound at the time of discharge showed appropriate SUB system function and decompressed renal pelvises and proximal ureteral dilation. The patient was discharged on the fifth day following general anesthesia and surgery. A final chemistry profile at the time of discharge showed persistently increased BUN of 70 mg/dL (18-39 mg/dL) and a creatinine of 6.7 mg/dL (0.7-2.0 mg/dL). No signs of respiratory distress were noted for the duration of the patient's hospitalization.

Following discharge the patient presented to the internal medicine service biweekly for the first two months after surgery and then twice over the next three months. These appointments were for re-evaluation of the bilateral SUB. The patient also presented once to the emergency critical care service two months postop for an episode of lethargy. During these rechecks, the patient's renal values remained persistently elevated with the BUN ranging from 43 to 122 mg/dL and the creatinine ranging from 3.7 to 9.3 mg/dL. The patient continued to show no signs of respiratory distress related to the previously diagnosed pneumothorax while in the hospital. Within the first four months postoperatively, the patient returned for multiple rechecks and eventual removal of the esophagostomy tube; no complications and no clinical signs of respiratory distress were noted at any subsequent reevaluations. After nine months of follow-up since the procedure, the owner reports no respiratory clinical signs.

## 3. Discussion

Most case reports describe cats presenting for spontaneous pneumothoraces due to underlying pulmonary pathology, but none describe primary bullous emphysema as a result of bullous rupture. This is the first case report to describe an asymptomatic suspected primary pneumothorax in a cat. Compared to other studies, findings of SPTs in cats included increased respiratory rate (100%) [11], abnormal lung sounds (63%-81%) [11, 12], and coughing (23%-29%) [11, 12]. This is also the first report of a suspected primary spontaneous pneumothorax in a cat. Previous reports of spontaneous pneumothoraces only report secondary SPTs [5–10]. Given the aforementioned presentation and diagnosis, this case report will serve to increase the diligent clinician's index of suspicion for SPTs in cats when interpreting radiographs and preparing for surgical interventions.

Primary spontaneous pneumothorax is a well-described pulmonary pathology in both humans and dogs, but there is limited research describing its incidence and treatment in cats. Interestingly, there are similarities between both people and dogs in clinical presentation, signalments, and outcomes. In humans, middle-aged ectomorphic males with deep chested conformations are overrepresented in cases of primary spontaneous pneumothorax [4]. The increased incidence of primary SPT in men with these descriptors is a result of increased forces at the apices of the lungs due to the conformation of their thoracic cages [15]. This is analogous to dogs with similar thoracic cage conformations. Incidents of primary spontaneous pneumothorax in dogs are most often reported in Siberian Huskies and other deep chested breeds due to similar conformation [3]. Although clear similarities exist between people and dogs, no such comparisons can be made regarding cats due to the paucity of information available.

The diagnosis of a pneumothorax is achieved with a combination of physical examination findings and imaging modalities. Regardless of its etiology, a correct diagnosis relies on a high index of suspicion based on respiratory signs, thoracic auscultation abnormalities, and radiographic findings consistent with a pneumothorax. Respiratory signs include asynchronous or inverse breathing patterns often coupled with increased respiratory rates. Thoracic auscultation is consistent with decreased lung sounds and has a sensitivity and specificity of 99% and 45%, respectively, in clinically affected patients [16]. Although thoracic auscultation provides a high degree of accuracy when diagnosing true positives, this becomes challenging when patients do not exhibit clinical signs. As emphasized with the case presented here, there is a need for meticulous case management even in patients that appear clinically normal. Without a comprehensive physical examination, and finding the enlarged kidney leading to an ultrasonographic renal evaluation and subsequent thoracic radiographs, this pneumothorax may have been missed. This also emphasizes the importance of performing presurgical thoracic diagnostic imaging studies in critical patients undergoing major surgical procedures, even with a low index of suspicion for intrathoracic pathology.

Radiographs are most frequently used as a cursory screening diagnostic for intrathoracic pathology. This is especially relevant with SPTs as one study showed that the sensitivity in dogs reached 100% [4]. In a more recent study, a right lateral horizontal beam projection was shown to have the highest sensitivity in detecting pneumothorax when compared to the VD/DV views [17]. Common radiographic signs include: retracted lung lobe margins, absence of pulmonary vasculature adjacent to the thoracic wall, sternum, and diaphragm, lack of sternal and cardiac silhouette contact, flattening of the diaphragm, and mediastinal shift. However, etiological confirmation was poor with only 17% of definitive causes diagnosed [18]. Additionally, detection of bullous emphysema and blebs ranged from 0 to 50% and potentially underestimates the severity of the lesions when present [4, 18]. Radiographs are an excellent survey diagnostic modality for determining the presence of pneumothorax, but lack of resolution and multidimensional imaging capabilities decrease their utility to determine its etiology. Therefore, thoracic computed tomography should be considered for surgical planning or definitive etiologic diagnosis in companion animals. There is only a 20% radiographic agreement between surgical findings and radiographs, and radiographs are less sensitive than CT for detection of pulmonary nodules [18, 19]. This conclusion was also reached in another study finding that CT is a better imaging modality compared to radiographs when determining the underlying cause of spontaneous pneumothorax in dogs [4]. This was especially evident in this case as the cat exhibited no clinical signs and thoracic radiographs showed no underlying cause for the pneumothorax. It was not until a CT was performed that the cause of the SPT was elucidated.

Another routine imaging modality is thoracic focused assessment with sonography for trauma (TFAST). This involves intercostal ultrasonography looking for decoupling of the visceral and parietal pleura; in normal patients these two tissue layers create a hyperechoic interface easily visualized with traditional linear probes. Interestingly, ultrasonography is superior to both auscultation and radiographs to diagnose pneumothorax in people and has a sensitivity of 78% and specificity of 93% in dogs [20, 21]. Although it can be also be used to determine the severity of the pneumothorax, its primary limitation is that it is technically demanding; experienced ultrasonographers can expect to improve the aforementioned sensitivity and specificity to 95% and 96%, respectively [21]. This specific modality was not used in this case as there was no clinical index of suspicion to investigate for intrathoracic pathology amenable to ultrasonographic imaging.

Computed tomography (CT) is the gold standard for diagnosing and evaluating SPT in people and has shown to have a positive predictive value of 92% in pediatric cases [22]. Its advantages over more traditional imaging options, such as radiographs, include: superior soft tissue resolution, differentiation of soft tissue densities based on Hounsfield units (HU), and multidimensional reconstruction capabilities. CT is shown to be greater than twice as effective in diagnosing SPTs in dogs versus radiographs with a correct etiological diagnosis of 75% versus 17% with radiographs [18]. Interestingly, a study in dogs showed that slice thickness, severity of the pneumothorax, and ventilation protocols did not affect the sensitivity, positive predictive value, or interobserver variability [23]. Although there is limited data available for pneumothorax in cats, a previous case report validated the utility of a CT scan in diagnosis of pulmonary blebs in cats with bronchodysplasia [10]. Given the extensive research in people, canine relevant publications and reports of its usage in cats, there is strong supporting evidence for the diagnostic utility of CT for identification of underlying etiology of feline SPT.

Evaluation of computed tomographic thoracic studies can be challenging to novice interpreters. Moreover, pneumothorax pathology further complicates the interpretation process for multiple reasons. First, concurrent loss of physiologic intrathoracic negative pressure results in atelectatic lungs. This finding can often be overinterpreted as primary pathology such as neoplasia or aspiration pneumonia. Second, based on the pathophysiology of a primary pneumothorax, air must escape from the pulmonary parenchyma into the thoracic cavity; this is through rupture of either bullae or blebs. However, the challenge lies in the fact that if air is escaping through bullae, for example, those bullae are no longer competent and their wall is ruptured allowing air to be released during the ventilatory process. Remembering that air provides an exceptional biologic contrast enhancement in CT studies, this air is no longer trapped within the bullae, which become deflated making diagnosis difficult. If multiple bullae exist, a diagnosis can still be made knowing that those evident on imaging are not the contributory bullae. However, if only one bulla exists, it is ruptured causing the pneumothorax, and with no other imaging findings to explain the pneumothorax (i.e., secondary causes) a diagnosis of exclusion of primary SPT can be suspected. Careful consideration must be given to the underlying etiology and variability in lesion numbers, as 83% of dogs have multiple lesions and 37% of dogs have multiple lung lobes affected [3, 4]. This is especially important for surgical planning where a complete evaluation of each lung lobe submerged in saline is necessary to locate the ruptured bullae.

Based on the thoracic radiographs at the time of intake, the initial discussions focused around potential iatrogenic introduction of air into the pleural space due to subcutaneous fluid administration and/or central venous catheter placement. However, this was prior to the computed tomographic study and emphysematous bullae diagnosis. The likelihood of an iatrogenic pneumothorax in this cat is considered less likely for multiple reasons. First, the radiographs showed subcutaneous emphysema located cranially and dorsally between the cat's scapula on midline. Anatomically, and based on radiographic measurements, it would be impossible for a 1.5 inch 22 gauge needle (the size of the needle used to administer fluids) to enter the pleural space in this overweight conscious animal without excessive discomfort. If this was the case, the needle would have penetrated through the subcutaneous tissues, epaxial musculature, traversed either abaxially or between the transverse spinal processes, to the dorsal ceiling of the intercostal musculature and into the pleural space. Furthermore, there was no evidence of either intrapleural fluid administration or subcutaneous emphysema tracts near the pleural space, both of which would be present should this SPT have been caused iatrogenically.

Placement of a central venous catheter (CVC) is another possible cause for iatrogenic pneumothorax since the CVC was placed before thoracic radiographs were obtained. Pneumothorax is a well-documented consequence of CVC placement in humans with an incidence of 1% to 6.6% [24] and is often referred to as a mechanical complication. Mechanical complications include arterial puncture, hematoma, hemothorax, pneumothorax, arteriovenous fistula, venous air embolism, nerve injury, thoracic duct injury (left side only), intraluminal dissection, and puncture of the aorta [25]. Mechanical complications are reported in approximately 5% to 19% of patients with pneumothorax representing about 30% of these complications [26, 27]. Unlike humans however, the true incidence of pneumothorax secondary to CVC placement in the veterinary literature is unknown. However, to the authors' knowledge, this complication is exceedingly uncommon and unreported in the small animal literature. For example, in a large study evaluating the use of CVC in dogs and cats, iatrogenic pneumothorax was not reported as a complication of central venous catheter placement [28]. In the same study, mechanical complications were the most frequently reported issue in the patient population and included most frequently the inability to draw blood back, followed by CVC displacement, suture breakage, kinking, infection, among others [28]. Another study including 27 dogs and 20 cats found that when comparing CVC placement with that noted in people, there were no serious complications during CVC placement in critically ill cats and dogs [29]. While exceedingly unlikely and very little evidence to support this theory, the authors do recognize that iatrogenic pneumothorax is possible. The bullous emphysematous disease noted on CT could have been an incidental finding, with no ruptured bullae present, and the pneumothorax was iatrogenic.

The authors also recognize that the presence of underlying lung pathology, with concurrent enraptured bullae, contributing to a secondary pneumothorax cannot be completely excluded. However, this is unlikely given the lack of any clinical or diagnostic imaging findings to support this theory. This is also unlikely given that the cat never became clinical either during the time of its treatment, throughout its entire surgical recovery period, or at recent follow-up conversation with the owner nine months after initial presentation. During this follow-up, the owners reported no new clinical signs attributable to alternative intrathoracic pathology. Furthermore, a secondary cause to the SPT could only be definitively determined with necropsy findings given the lack of radiographic evidence to focus in vivo sampling efforts. However, given that the lower limit of detection of pulmonary pathology on a CT reaches lesions measuring 1 mm in diameter [30, 31], it is unlikely that such a small area of underlying pathology would contribute to a secondary pneumothorax in this case. This is especially relevant knowing that multiple bullae were found on the CT imaging study giving the diagnostician a primary reason for the intrathoracic air. Also of note, the suspicion of primary SPT in people is most often based on the patient's history [32, 33]. The diagnosis is then confirmed with thoracic CT findings of bullous emphysema that lack any concurrent evidence of underlying lung disease [32, 33]. Even the classification of primary and secondary SPT in people is subject to change in the future due to the increasing number of human patients diagnosed with primary SPT found to have subtle undiagnosed pulmonary abnormalities [33]. While veterinary medicine has traditionally relied on pathologic confirmation of bullae/blebs, this mandates either surgical intervention or necropsy for sampling. This may not be necessary in some cases, contraindicated in others, such as the one described here, where clinical signs are mild and future monitoring is all that is needed for long-term management.

Initial triage and stabilization of clinical SPT involves oxygen supplementation, thoracocentesis, or thoracostomy tubes, which were not necessary in this case due to the lack of clinical signs and absent respiratory compromise. The lack of clinical signs and bullae diagnosis is especially important to note in this case regarding general anesthesia. With no clinical signs associated with the SPT, there was no reason to suspect bullous emphysema. In select cases, barotrauma from normal positive pressure manual ventilation can result in alveolar and pleural rupture, exacerbated by concurrent bullae or blebs, as the surrounding parenchyma is compromised [34–36]. This can become a life-threatening intraoperative complication if not recognized quickly secondary to routine positive pressure ventilatory technique during anesthetic events.

Medical management of pneumothoraces is highly dependent on the severity of the underlying pathology and patient's stability ranging from 73% to 100% failure rate and a 50% recurrence rate [3, 4, 37, 38]. This is in direct contrast to people where the failure rates are much lower ranging from 16% to 50% [39]. Surgery is therefore often indicated with SPTs and is the gold standard in people with a 1.5% recurrence rate [40]. Surgical interventions have an 88% success rate in dogs and involve removing the compromised region of lung parenchyma through either a median sternotomy, lateral thoracotomy, or thoracoscopic technique [3, 37, 41]. There is some debate surrounding the treatment of SPTs in cats due to the lack of research describing the outcomes of surgical versus medical therapy. The consensus for SPTs in cats has been extrapolated largely from the canine literature utilizing a combination of medical and surgical intervention where there exists a decreased recurrence and mortality rates of dogs treated surgically versus medical management alone [3]. One study evaluating the treatment of SPTs in cats showed that surgical correction was associated with a poor outcome, but this association was most likely due to the severity and diffuse nature of the disease and should not predicate recommendations for medical management alone [11]. In another case report describing SPTs in a cat, the patient was treated successfully with partial lung lobectomies of the right middle, right cranial, and left cranial lung lobes with no complications postoperatively or at the two-month recheck [10]. For the case presented in this report, the pneumothorax was mild enough that neither conservative (thoracocentesis, thoracostomy tubes, continuous suction, and pleurodesis) or surgical interventions were indicated for treatment. Intense medical monitoring was elected with a long-term successful outcome. This illustrates that with mild pneumothorax in patients exhibiting no clinical signs, simple monitoring without procedural interventions can be pursued with a favorable long-term prognosis.

This case study has many implications for feline clinicians and future studies of feline spontaneous pneumothoraces. First, this case reported the first primary SPT in cats. It also illustrated the possibility of a patient with no clinical signs, yet having a potentially lethal comorbidity that could manifest during general anesthesia. Second, the utility of using CT in the diagnosis of primary SPT in cats was also elucidated, and it should continue to be used by clinicians for this purpose and surgical planning. Finally, there is a need for more feline SPT cases to be reported in the future allowing for treatment recommendations rather than extrapolated from people and canine studies.

## 4. Take-Home Messages

- Primary spontaneous pneumothorax can occur in cats and should be included in the differential list of cats presenting with respiratory distress
- Definitive primary spontaneous pneumothorax can be diagnosed in small animal species with appropriate clinical history, thorough physical examination, and computed tomography
- It is possible for small animal patients to present with nonclinical spontaneous pneumothorax. Clinicians should be aware of this presentation when considering potential anesthetic complications associated with bullae rupture, which may not always be diagnosed prior to surgery or anesthesia

## Data Availability

The data used in this case study can be provided on demand from the corresponding author upon request.
